# Quantifying topical antimicrobial use before and during participation in an antimicrobial stewardship programme in Dutch companion animal clinics

**DOI:** 10.1371/journal.pone.0283956

**Published:** 2023-04-13

**Authors:** Nafsika Kardomatea, Nonke E. M. Hopman, Ingeborg M. van Geijlswijk, Lützen Portengen, Jaap A. Wagenaar, Dick J. J. Heederik, Els M. Broens

**Affiliations:** 1 Department of Biomolecular Health Sciences, Faculty of Veterinary Medicine, Utrecht University, Utrecht, The Netherlands; 2 Department of Population Health Sciences, Faculty of Veterinary Medicine, Utrecht University, Utrecht, The Netherlands; Universidade Nova de Lisboa, PORTUGAL

## Abstract

The emergence of bacterial strains resistant to topical antimicrobials in both human and veterinary medicine has raised concerns over retaining the efficacy of these preparations. Yet, little information is available regarding the use of topical antimicrobials in either sector for planning targeted interventions. This study aims to quantify the use of topical antimicrobials in 44 Dutch companion animal clinics before and during their participation in an antimicrobial stewardship programme (ASP), to explore the effect of the intervention on topical antimicrobial use (AMU). Hence, prescription and clinic animal population data, collected from July 2012 until June 2018 were used. Specifically, the period from July 2012 until June 2015 was defined as pre-intervention period, whereas clinics started to participate in the ASP from March 2016 onwards. As quantification metric, the Defined Daily Dose for Animals (DDDA) was used and a mixed effect times series model with auto-regression was applied to monthly topical AMU data. The intervention effect was modelled using a step function with a change in (linear) time trend and clinic characteristics, as potential determinants of topical AMU, were assessed using a multivariable regression model. A seasonal pattern was identified, in the pre-intervention period, where topical AMU was highest in July-August and lowest in February-March. In addition, total topical AMU appeared to significantly decrease over time in the pre-intervention period and the proportion of dogs in the clinic was positively associated with topical AMU. The intervention effect was significant only for second line and for skin product AMU. This study demonstrates that during participation in an ASP, second line and skin product AMU decreased in Dutch companion animal clinics. Additionally, this study demonstrates the existence of a seasonal effect and a decrease in topical AMU over time already before introduction of a targeted intervention.

## Introduction

Topical antimicrobials (TAMs) are used in human and veterinary medicine to treat various infectious conditions either as monotherapy or in combination with systemic antimicrobials [1, 2]. TAMs in human medicine are primarily used to treat dermatological conditions, bacterial infections of the nose and eyes, as well as preoperatively or during ventilation in Intensive Care Units for decolonization purposes [1]. In companion animals, TAMs are commonly used to treat ear, skin and eye infections [2–4]. Their use might be preferred by practitioners over systemic antimicrobials mainly because of three characteristics; their ability to reach higher concentrations at the site of infection, less systemic side effects (including selection for antimicrobial resistance in the gut) and the possibility to use antimicrobial agents with low bioavailability like aminoglycosides [1].

Yet, some drawbacks are associated with the use of TAMs. Robust clinical efficacy data and clinical breakpoints for topical use are currently lacking in both human and veterinary medicine, although efforts are being made in that direction [5, 6]. Besides the possibility of emergence of antimicrobial resistance (AMR) at the application site, an additional risk of TAMs is that not only the patient but also the applicant and the environment may be exposed to the antimicrobial.

Any use of antimicrobials selects for antimicrobial resistance [7]. This has been extensively described for systemic use of antimicrobials, but for TAMs this is not yet fully elucidated [7]. In human medicine, several studies have reported the emergence of multi-drug resistant (MDR) *Staphylococcus aureus*, including methicillin-resistant *Staphylococcus aureus* (MRSA), after the use of fusidic acid and mupirocin [7, 8]. Retaining the efficacy of mupirocin is crucial since it is the sole topical antimicrobial approved for the decolonization of nasal MRSA in humans [9]. Likewise, fusidic acid efficacy should be retained, since it is also one of the final options to treat bacteremia caused by MRSA in humans [10].

In companion animal veterinary medicine, mupirocin is not approved for use in Europe, contrary to the United States [11]. Contrastingly, fusidic acid is not recommended for use in companion animals in the United States, but it is approved for topical use in Europe [10]. Despite a limited number of products approved for use in veterinary medicine containing these antimicrobials, fusidic acid and mupirocin resistant *Staphylococcus pseudintermedius* strains have been isolated from dogs [11, 12]. A potential explanation for this might be that point mutations appear to be sufficient to drive resistance regarding these two antimicrobials [13, 14].

To date, mitigation strategies regarding antimicrobial use (AMU) and AMR have focused on systemically administered antimicrobials adopting a One Health approach, thus engaging both the human and animal sector [1, 15]. One pillar of this approach is the establishment and implementation of antimicrobial stewardship programmes (ASPs) [16]. These programmes aim to ensure positive patient outcomes, whilst reducing the inadvertent effects of AMU, which also encompass the development of AMR [17]. While ASPs are implemented in an increasing number of human hospitals across several countries, the implementation of such programmes in veterinary settings is limited [18, 19]. A crucial element of ASPs is the quantification of AMU, which gives insight, provides information on changes over time, facilitates benchmarking and enables the evaluation of the impact of mitigation measures [20]. Most commonly, the antimicrobial consumption metric that is used in human medicine is the Defined Daily Dose (DDD) [20]. This metric has been adjusted to measure AMU in livestock and is used in antimicrobial consumption reports in veterinary medicine [21]. Recently, this metric, called Defined Daily Dose for Animals (DDDA) was applied in companion animal clinics in the Netherlands in order to quantify systemic AMU [22]. The metric was also applied in the Antimicrobial Stewardship and Pets (ASAP) project in which the authors developed and evaluated an ASP intervention in Dutch companion animal clinics [23, 24].

So far, no standardized quantification metric has been established for topical AMU in either the human or the animal sector. A standardized quantification metric could provide insight into topical AMU prescribing patterns, reveal the potential interplay between topical and systemic AMU and potentially contribute to minimizing AMR. The aim of the present study was to quantify topical AMU in 44 Dutch companion animal clinics before and during their participation in an ASP, to explore the effect of participating in the ASP on topical AMU.

## Materials and methods

### Study design

Data collected during the former ASAP-project were used [23, 24]. AMU data were retracted from the Practice Management System from 44 Dutch companion animal clinics. The full dataset of the ASAP-project included monthly topical and systemic antimicrobial prescription data as well as clinic animal population data from 44 Dutch companion animal clinics in the period from July 2012 until June 2018. In 2015, an antimicrobial stewardship programme (ASP) was developed and subsequently introduced stepwise in these clinics. The ASP aimed at increasing awareness on AMU, decreasing total AMU whenever possible, replacing systemic treatment with topical treatment (with or without antimicrobials) when indicated and shifting AMU towards first line antimicrobials, according to present national AMU guidelines and policy [S1 Table; 24]. The ASP consisted of different intervention elements, and therefore the actual intervention period comprised 12 months, from the start of the implementation of the ASP up to 4–5 months after the last element. The participating clinics were timewise divided into four clusters based on their geographical location. The intervention period of the first cluster started in March 2016, the intervention period of the last cluster started in January 2017. The applied stepped-wedge design and the time schedule is available at https://doi.org/10.1371/journal.pone.0225124.g001 [24]. Overall, the study period ranged from July 2012 until June 2018 and was divided in two periods; the pre-intervention period (July 2012-June 2015, comprising three periods from July of the previous year to June of the next year) and the intervention period, as previously described.

### Calculation of topical AMU

To quantify topical AMU, the number of Defined Daily Doses for Animals (DDDA) was used in a similar way to the quantification of systemic AMU, which was applied in previous studies [22–24]. The metric was adjusted for TAMs. In summary, DDDA for TAMs is calculated as the quotient of a fraction in which the numerator is the number of packages of a specific TAM product that was dispensed by a clinic in a defined time period multiplied by the Defined Daily Dose (DDD) assigned for this specific TAM product. In the case of TAMs, the DDD is equal to the assumed average duration of treatment. The assumed average duration of treatment is the number of days that the product should be applied to the companion animal to complete the treatment. This duration was extracted from the Standard Product Characteristics (SPC) of each TAM, when available, or an assumption was made based on expert opinion. The DDDs were specified for each TAM product that was dispensed by the clinics present in the dataset. The denominator was the animal population that attended the clinic in a predefined time period (of three years), which in this study consisted of the sum of the number of dogs, cats and rabbits. Thus, the formula of the DDDA adjusted for TAMs is the following:

$$DDDA=\frac{Number\;of\;packages\;dispensed\times DDD\;per\;product}{Animal\;population}$$ [1]

For each clinic (if data were available), topical DDDA was calculated per month from July 2012 until June 2018. The mean total topical AMU was estimated for each period of the study based on the data from the participating clinics and was expressed as DDDA/year.

Antimicrobials were categorized in two ways: per application category (e.g., ear, eye and skin) and per antimicrobial category (1^st^ line, 2^nd^ line or 3^rd^ line, according to the Dutch guidelines and policy on veterinary AMU; Table 1) [25].

**Table 1:** Classification of veterinary AMU according to Dutch policy on veterinary AMU [25].

Classification	Reasoning	Main classes of AMs
**1**^**st**^ **line**	Empirical therapy; Do not select for (to current knowledge), nor are specifically meant for treatment of ESBL-producing micro-organisms.	Tetracyclines, nitroimidazoles, narrow-spectrum penicillins, trimethoprim, sulfonamides, lincosamides and phenicols.
**2**^**nd**^ **line**	All AMs not classified as 1^st^ or 3^rd^ choice AMs; Use of these AMs might select for ESBL-producing bacteria or is specifically indicated in case of an ESBL-infection.	Aminopenicillins (with/without beta-lactamase inhibitors), 1^st^ and 2^nd^ generation cephalosporins, aminoglycosides and polymyxins.
**3**^**rd**^ **line**	A selection of Highest Priority Critically Important AMs for human medicine according to WHO; By Dutch law restricted to use only in individual animals and after culture and susceptibility testing.	Fluoroquinolones, 3^rd^ and 4^th^ generation cephalosporins.

### Statistical analysis

#### Pre-intervention period

Pre-intervention data (July 2012 –June 2015) were used to explore time trends, seasonality, and potential determinants for topical AMU in a similar way as described before [23]. The pre-intervention period comprised of three periods, each spanning from July of the previous year to June of the next year (i.e. first period; July 2012-June 2013, second period; July 2013-June 2014, third period; July 2014-June 2015). In short, time trends were modelled using natural regression splines with a single interior knot placed at the median time (January 2014), while seasonality was modelled using a combination of two pairs of harmonic functions. Clinic-specific intercepts and trend coefficients as random effects and an auto-regressive (AR1) correlation structure for the residuals were included. The models estimate geometric mean (GM) topical AMU. Additionally, Geometric Mean Ratios (GMRs), which are the ratios between two GMs, were utilized to quantify covariate effects. Finally, average exposure was also estimated for each period (i.e. year; from July of one year to June of the next year) separately, using the raw pre-intervention data and expressed as DDDA/year.

Clinic characteristics were assessed as potential determinants of topical AMU using a multivariable regression model with log-transformed topical AMU data as dependent variable. As the number of veterinarians per practice was highly correlated (Pearson’s r = 0.75) with the total number of dogs, cats and rabbits, this variable was excluded from subsequent analyses to reduce collinearity. Similarly, because the proportion of cats was highly correlated (Pearson’s r = 0.75) with the proportion of dogs, it was also excluded from subsequent analyses for the same reason. P-values equal to or smaller than 0.05 were considered statistically significant.

#### Effect of participation in the ASP

Data from 12 months before introduction of the ASP until 12 months after introduction of the ASP were used to evaluate the intervention effect on topical AMU. Statistical analysis was again similar as described before [24]. In short, a mixed effect time series model that allowed for a linear trend over time was used to describe monthly topical AMU from 12 months before until 12 months after introduction of the ASP. Seasonality was modelled using Fourier terms (sine and cosine). Topical AMU appeared to follow an approximate log-normal distribution. Consequently, log-transformed topical AMU data were used as the dependent value. Zero values were substituted with 2/3 of the lowest recorded use before log-transformation. The (sole) intervention effect was modelled using a step function and by modelling a change in the (linear) time trend during the intervention period to that before the intervention period. The estimated average intervention effects across clinics are presented as GMRs and as proportional decreases in use.

The organization of the datasets and the visualization of the outcomes were conducted using the programming language R in “RStudio” and the package “nlme” (version 3.1) was used for the main statistical analyses.

## Results

### Pre-intervention period

In total, 44 clinics provided monthly topical antimicrobial prescription data between July 2012 and June 2015. Based on the available data, 62,282 topical antimicrobial packages were prescribed overall during this period. Out of those, 19,532 (31.4%) concerned first line TAMs and 36,290 (58.3%) concerned second line TAMs. Regarding the product types, 27,492 (44.1%) out of the total number of packages prescribed, concerned ear products while 23,902 (38.4%) concerned eye products.

### Mean topical AMU per year, time trend and seasonality (pre-intervention)

**Fig 1:**
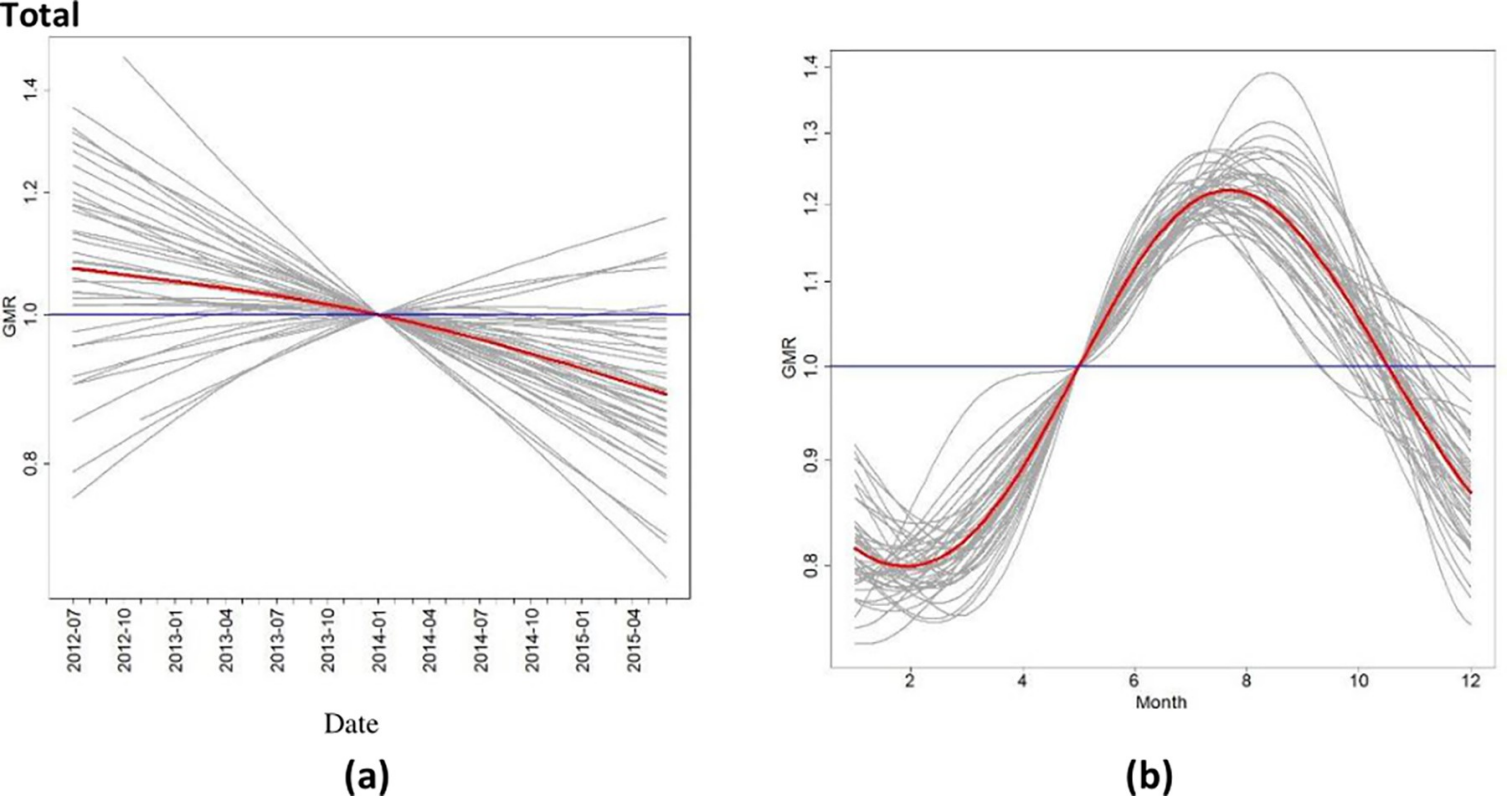
Results of the statistical model regarding the pre-intervention period (July 2012-June 2015). (a) Time trend over the months with y-axis displaying the geometric mean ratio (GMR) and x-axis the date (year and month). Time trends were modelled using natural regression splines. The GMR compares the geometric mean (GM) of topical AMU in each month to the average GM across the three-year period (GMR = 1 on January 2014 by definition). The grey lines depict the clinic-specific estimates, whereas the red line depicts the mean of the random effects distribution. Time trend is displayed for total topical AMU. (b) Seasonal pattern of total topical AMU. In the y-axis the GMR is displayed and on the x-axis the months. The seasonal effect was modelled using harmonic functions. The GMR compares the GM of topical AMU in each month to the average GM across the year (GMR = 1 on the month of May by definition). The grey lines depict the clinic-specific estimates, whereas the red line depicts the mean of the random effects distribution.

The statistical model indicated a statistically significant decrease in total topical AMU over time from 0.93 DDDA/year in the first period (July 2012-June 2013) to 0.84 DDDA/year in the third period (July 2014-June 2015) (Fig 1(A)). Use of first line ear and eye products also decreased significantly over time. Use of second line products showed no change. For the other categories, data were too limited for statistical analysis.

A strong seasonal effect was observed in total topical AMU, with the highest use in July-August and lowest in February-March (Fig 1(B)). A similar pattern was observed for first and second line topical AMU, as well as for ear and eye products. Again, for the other categories data were too limited for statistical analysis.

### Determinants of topical AMU (pre-intervention)

The multivariable regression analysis of potential determinants of topical AMU showed that only the proportion of dogs was significantly associated with topical AMU (Table 2). Clinics with a larger proportion of dogs tended to have a higher total topical AMU (GMR 1.18, 95% CI 1.04–1.34 per 10% increase in the proportion of dogs) implying that with each 10% increase in the proportion of dogs the ratio of geometric means of total topical AMU increases by 18%. This was also significant for first and second line topical AMU, as well as for ear products (GMR for first line topical AMU 1.17, 95% CI 1.01–1.35, GMR for second line topical AMU 1.18, 95% CI 1.04–1.34, and GMR for ear product AMU 1.24, 95% CI 1.05–1.46, per 10% increase in the proportion of dogs, respectively). For eye products this was only borderline significant (GMR 1.14, 95% CI 1.00–1.29, per 10% increase in the proportion of dogs). The remaining clinic characteristics displayed no statistically significant associations with topical AMU or were only borderline significant.

**Table 2:** Effect estimates for potential determinants of topical AMU. The results are the output of a multivariable regression model for total, first line, second line topical AMU and ear and eye product AMU. The data are log-transformed.

	Total		First line		Second line			Ear		Eye	
Clinic characteristics	GMR	95% CI	GMR	95% CI	GMR		95% CI	GMR	95% CI	GMR	95% CI
Proportion of dogs (per 10% increase)	1.18	**1.04–1.34** *	1.17	**1.01–1.35** *	1.18	**1.04–1.34** *		1.24	**1.05–1.46** *	1.14	1.00–1.29**
Proportion of rabbits (per 1% increase)	0.95	0.90–1.00**	0.98	0.93–1.04	0.95	0.90–1.00**		0.95	0.88–1.01	0.98	0.93–1.03
Total number of animals (per 1000)	0.95	0.88–1.01	0.94	0.86–1.02	0.95	0.88–1.01		0.92	0.84–1.01**	0.97	0.90–1.04
Number of affiliated practices	1.13	0.91–1.40	1.21	0.94–1.55	1.13	0.91–1.40		1.08	0.81–1.43	1.12	0.90–1.39
Mean experience per clinic (per 10 years)	0.97	0.83–1.14	0.93	0.77–1.12	0.97	0.83–1.14		0.89	0.72–1.10	0.98	0.83–1.15
Companion animals only	0.79	0.52–1.21	0.97	0.60–1.56	0.79	0.52–1.21		0.9	0.52–1.57	0.81	0.53–1.24
Urban (versus rural or mixed)	1.15	0.86–1.55	1.15	0.82–1.60	1.15	0.86–1.55**		1.29	0.88–1.88	1.1	0.82–1.47
Conventional medicine only	1.21	0.86–1.70	1.34	0.91–1.97	1.21	0.86–1.70		1.02	0.65–1.58	1.22	0.87–1.71
Graduated in Utrecht	1.18	0.91–1.54	1.1	0.81–1.49	1.18	0.91–1.54		1.16	0.82–1.64	1.2	0.92–1.56
Female veterinarians only	1.06	0.78–1.43	0.98	0.69–1.39	1.06	0.78–1.43		0.96	0.65–1.43	1.1	0.81–1.49
Not serving shelters/kennels	0.88	0.69–1.13	0.94	0.71–1.25	0.88	0.69–1.13		0.8	0.58–1.11	0.95	0.74–1.21
Not serving breeders	1.05	0.76–1.44	0.94	0.65–1.34	1.05	0.76–1.44		1.1	0.73–1.65	1.07	0.78–1.46
PMS 2^1^ (versus 3 others)	1.05	0.78–1.42	1.19	0.84–1.68	1.05	0.78–1.42		1.04	0.70–1.54	1.13	0.83–1.52

^1^ PMS = Practice Management System type 2

*P-value ≤ 0.05

**0.05< P-value ≤0.10

### Intervention effect

Based on the data, the total number of packages of topical antimicrobials prescribed during participation in the ASP was 31,588; 14,614 (46.3%) concerned first line TAMs and 16,314 (51.6%) concerned second line TAMs. Regarding product types, 13,825 (43.8%) packages that were prescribed concerned ear products, 12,445 (39.4%) concerned eye products and 4,809 (15.2%) concerned skin products.

**Fig 2:**
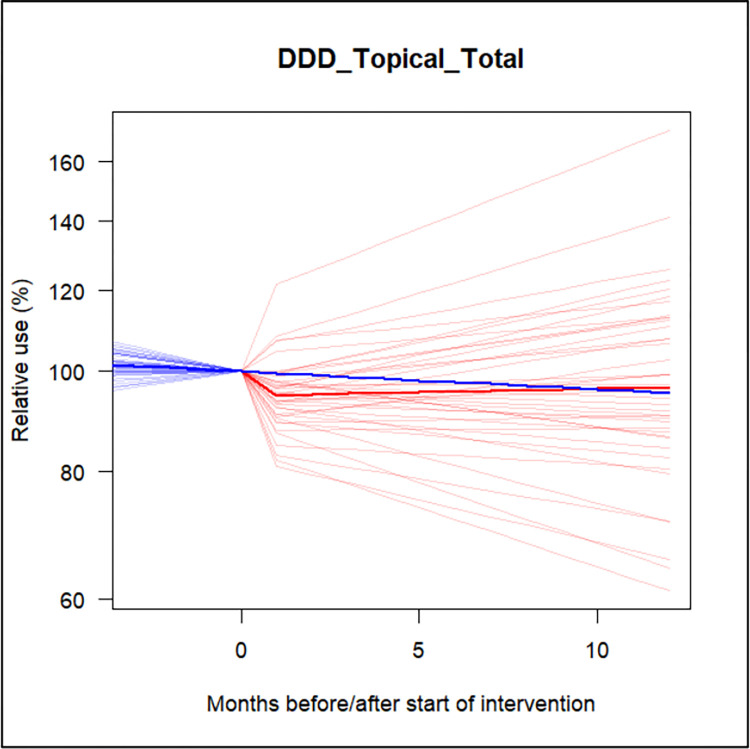
Average (bold lines) and clinic-specific (thin lines) of total topical antimicrobial use. The blue line indicates the trend if no intervention had occurred. The red lines indicate the effects of the intervention. This is based on the estimation of the model of the trend, drop and slope regarding total topical antimicrobial use.

The models suggest little evidence for a strong and/or sustained effect of the intervention (on top of the already ongoing time trends) for most topical AMU categories, except perhaps for second line and skin product AMU, which appear to significantly decrease shortly after introduction of the ASP. However, this effect does not appear to be sustained thereafter (S2 and S5 Figs). Additionally, the estimated between-clinic variation in intervention effect appeared to be substantial, meaning that it would be difficult to predict the effect of participation in the ASP on topical AMU for an individual clinic (Fig 2).

## Discussion

The main objective of this study was to assess the impact of participation in an antimicrobial stewardship programme (ASP) on topical AMU in 44 Dutch companion animal clinics by quantifying topical AMU before and during participation in this ASP. Furthermore, trends of topical AMU and seasonality were explored in the pre-intervention time period. This study was performed using data from the former ASAP-project, which evaluated the impact of participation in an ASP on systemic AMU in the same companion animal clinics and over the same period [23, 24]. Importantly, the quantification metric that was used in both studies was the same (i.e. number of DDDA) [23, 24]. The study period ranged from July 2012 until June 2018 and was divided in two periods; the pre-intervention period (July 2012-June 2015, comprising three periods from July of the previous year to June of the next year) and the period when clinics participated in the ASP. Because of the stepped-wedge design of the ASAP-project, not all companion animal clinics were introduced to the ASP at the same time [24]. Participation of individual clinics in the intervention programme took place between March 2016 and March 2018.

In the pre-intervention period, a significant change in total topical AMU was detected. Particularly, the use decreased from a mean of approximately 0.93 DDDA/year (in 2012–2013) to 0.84 DDDA/year (in 2014–2015). A comparable significant decrease was observed in the same period for systemic AMU from 1.82 DDDA/year to 1.56 DDDA/year [23]. This could be explained by the fact that awareness regarding AMU and AMR had already been increased in the Netherlands and policies aiming to reduce AMU were implemented [26].

Based on the outcomes of the present study, topical AMU constitutes a substantial part of total AMU. The mean total topical AMU, expressed in number of DDDAs, is approximately 50% of the estimated total systemic AMU in the same clinics and period. This ratio is in line with recent observations by Méndez and Moreno, who reported 5.3 and 8.1 DDDAs per year for topical and systemic AMU respectively, in a sample of dog patients (n = 242) of 10 companion animal veterinary clinics in Madrid, Spain [27], although extrapolated from the collected number of treatments. Earlier, Singleton et al. had reported similar proportions of topical AMU (i.e. 7.4% topical and 12.2% systemic antimicrobial agent prescriptions in dogs) with yet another quantification metric, prescription rate during visits, for their estimations [28]. Contrastingly, a lower proportion of topical AMU was reported by Mateus et al. using a similar quantification metric (i.e. 22.6% topical and 77.4% systemic antimicrobial administrations or prescriptions in dogs) [29]. Topical AMU might contribute to AMR as well. Recognizing it constitutes about one third of total AMU in companion animals, topical AMU should be included when aiming for optimization of veterinary AMU.

A significant seasonal effect was discovered for total topical AMU and the same was observed for first line, second line, ear and eye product AMU, before the ASP was introduced. The use peaked in the months of July-August and was lowest in the months February-March. Hopman et al. reported a similar seasonal pattern for systemic AMU [23]. In their study, they mentioned that highest use might be observed in warmer months, because certain conditions display a seasonality in their occurrence, such as allergic dermatitis, which is observed more frequently in warmer months [23, 30]. In addition, it is mentioned that in summer months, injuries from bite wounds or other dermatological issues might be more frequent [23]. This could also explain the seasonal pattern that was found in the present study, regarding topical AMU since TAMs are applied in dermatological conditions and in cases of otitis [2, 31]. Furthermore, in warmer months, owners and their pets might spend more time outdoors engaging in activities such as swimming in open water, which might predispose dogs for ear infections [32].

The proportion of dogs in the clinics was significantly and positively associated with total topical AMU. This was also the case for first line, second line and ear product AMU. Thus, clinics with a higher proportion of dogs seemed to use more TAMs. In this study, the proportion of cats was excluded from the statistical analysis, because of its high (inverse) correlation with the proportion of dogs. Therefore, this covariate could not explicitly be evaluated as a potential determinant of topical AMU. Nevertheless, it is important to note that, in this case, a higher proportion of dogs means a lower proportion of cats, which could provide some insight into this covariate’s potential relation to topical AMU. Two British studies also reported that dogs were prescribed more topical antimicrobial agents compared to cats [28, 29]. Singleton et al. argued that this might be explained by a better compliance of dogs regarding TAM applications and by the fact that dogs have a higher prevalence of certain dermatological diseases compared to cats [28]. These factors might explain the observed results in the present study as well.

No significant effect was observed in total topical AMU that could be attributed to participation in the ASP. This could be explained by the fact that while second line topical AMU significantly decreased during participation in the ASP, first line topical AMU increased, albeit not significantly. Therefore, total topical AMU appeared not to decrease significantly overall. Another factor that might have contributed to this outcome is the substantial between-clinic variation that was observed after introduction of the ASP. Besides, total topical AMU already showed a decreasing time trend in the pre-intervention period.

The aims of the ASP were to increase awareness on AMU, decrease total AMU whenever possible, replace systemic treatment with topical treatment (with or without antimicrobials) when indicated and shift AMU towards first line antimicrobials, according to Dutch guidelines and policy on veterinary AMU [Table 1; 25, 33]. Hopman et al. reported that regarding systemic AMU, a statistically significant decrease in first and second line AMU was observed, with a shift towards first line AMU, that could be attributed to participation in the ASP [24]. Similar trends were observed in the present study for topical AMU as well, but less clear. Nevertheless, the notable variation between clinics with regard to topical AMU, indicates that there are still possibilities for improvement concerning categories of TAM used.

Interestingly, ear product AMU demonstrated an initial drop after introduction of the ASP. However, over time it increased again, surpassing the estimated AMU had no intervention occurred. The initial drop in ear product AMU might be slightly exaggerated because of the choice of the statistical model that was used in the study. Specifically, the intervention was modelled as a combination of an initial drop in use and a linear correction on the pre-intervention time trend. This is a rather crude approximation to the more complex effects that may be seen in reality and the initial drop in use should be interpreted cautiously as it may equally appear from an initial slow decrease followed by an increase. However, as mentioned in the paper by Hopman et al., the goal of the ASP was to optimize AMU [24]. To that end, certain treatment guidelines were suggested during the ASP, which indicated that topical AMU is preferred over systemic AMU in cases of otitis externa [24, 25, 33, 34]. Therefore, it can be surmised that the observed increase in ear product AMU might be attributable to this optimization on AMU, which may additionally constitute a plausible reason for why total topical AMU appeared not to decrease during participation in the ASP.

The strengths and weaknesses of the present study are largely identical to those described in the study on systemic AMU, since the study design and statistical analyses are the same [24]. The present study had the added advantage of using data from the ASAP-project [23, 24] and using a similar quantification metric, which made it possible to observe the changes in both systemic and topical AMU in the same clinics. Therefore, a preliminary assumption on the potential interplay between systemic and topical AMU could be made, which could be a starting point for future research on the subject. Furthermore, the estimation of topical AMU regarding different product categories, gives more insight on prescribing patterns of the companion clinics over time and in different seasons. Concerning the study design, a strength of this study was the repeated monthly measurements for each participating clinic. This enabled to control the possible intervention effects for time trends occurring in parallel, meaning that the influence of other factors on the observed effect of participation in the ASPs was less probable. However, the present study did not explore the appropriateness of topical AMU since animal patient-specific information was not available for these 44 companion animal clinics nor did it explore the dosing regimens of the TAMs prescribed. This constitutes a limitation since, overall (topical) AMU is a non-specific measure without further information on appropriateness of antimicrobial therapy or data on patient outcomes. Moreover, a quantitative reduction in (topical) AMU does not necessarily mean an increase in the quality of prescribing. Besides, and specifically looking at TAMs, it is often difficult to determine and monitor the exact dosing [35]. However, since any use of antimicrobials might select for AMR, any reduction that can be reached and a shift towards antimicrobials of lower importance, by improving awareness on AMU and adherence to current guidelines is an advantage. A future recommendation would be to incorporate these aspects on quality of use, and explore which, how much and how many TAMs are applied regarding specific clinical conditions and (preferably) clinical outcomes, to be able to evaluate quality of antimicrobial prescribing. Another limitation of the present study is the possibility that participating veterinarians could have changed their antimicrobial prescribing behaviour, already before the start of the ASP, because veterinary clinics were contacted two to three months in advance and because of pre-existent antimicrobial interest and focus. Consequently, the effect of participation in the ASP might be somewhat diminished. Besides, the stepped-wedge design of the study might have allowed for clinics in which the ASP was already introduced, to share information with clinics, which were still in the baseline period. Yet, this effect was expected to be minimal, since the clinics that participated in the ASP were grouped on a geographic location basis.

The representativeness of participating clinics for the entire country might be questioned, since the participating 44 clinics were selected based on willingness to participate, which is opposite to random selection. Therefore, prescribing behaviour may have already been more prudent and motivation to change antimicrobial prescribing behaviour might have been higher compared to other, not-participating clinics. Still, the results of this study do offer an initial overview of topical AMU patterns in companion animal clinics, which despite the aforementioned limitations, could constitute a starting point for future research to explore and shed more light on an, until recently, often overlooked veterinary antimicrobial prescribing area.

## Conclusion

The analyses and quantification of topical AMU in 44 Dutch companion animal clinics over a three-year period showed a seasonal effect and a decrease in topical AMU over time. Moreover, the proportion of dog patients in the clinics showed a positive association with topical AMU. During participation in an ASP, second line topical AMU decreased, whereas first line topical AMU appeared to increase, which was in line with the goals of the ASP. Likewise, ear product AMU appeared to increase over time after introduction of the intervention, which may suggest a shift from systemic AMU to topical AMU in the treatment of ear infections. Overall, this study, using DDDA as quantification metric to quantify topical AMU, could serve as a first step towards elucidating topical AMU patterns in companion animal clinics and to assess the influence of antimicrobial stewardship interventions on topical AMU in veterinary medicine.

## Supporting information

S1 Table Separate intervention elements as offered during the ASP. The time point when these elements were offered as well as who were involved and the estimated time investment are displayed. “S-team”refers to a Support-Team that was assembled for the ASP and is comparable to the human Antibiotic Stewardship-Teams (A-teams). The S-team was comprised of a veterinary microbiologist, a veterinary specialist in internal medicine of companion animals, a veterinary pharmacologist, a hospital pharmacist and the project leader.
(TIF)

S1 Fig Intervention effect regarding 1^st^ line topical AMU. (TIF)

S2 Fig Intervention effect regarding 2^nd^ line topical AMU. (TIF)

S3 Fig Intervention effect regarding ear product AMU. (TIF)

S4 Fig Intervention effect regarding eye product AMU. (TIF)

S5 Fig Intervention effect regarding skin product AMU. (TIF)
