# Supplementary figures and images for: Quantifying topical antimicrobial use before and during participation in an antimicrobial stewardship programme in Dutch companion animal clinics
Source: PLoS One. 2023 Apr 13;18(4):e0283956. doi: 10.1371/journal.pone.0283956 (PMC10101466; doi:10.1371/journal.pone.0283956)

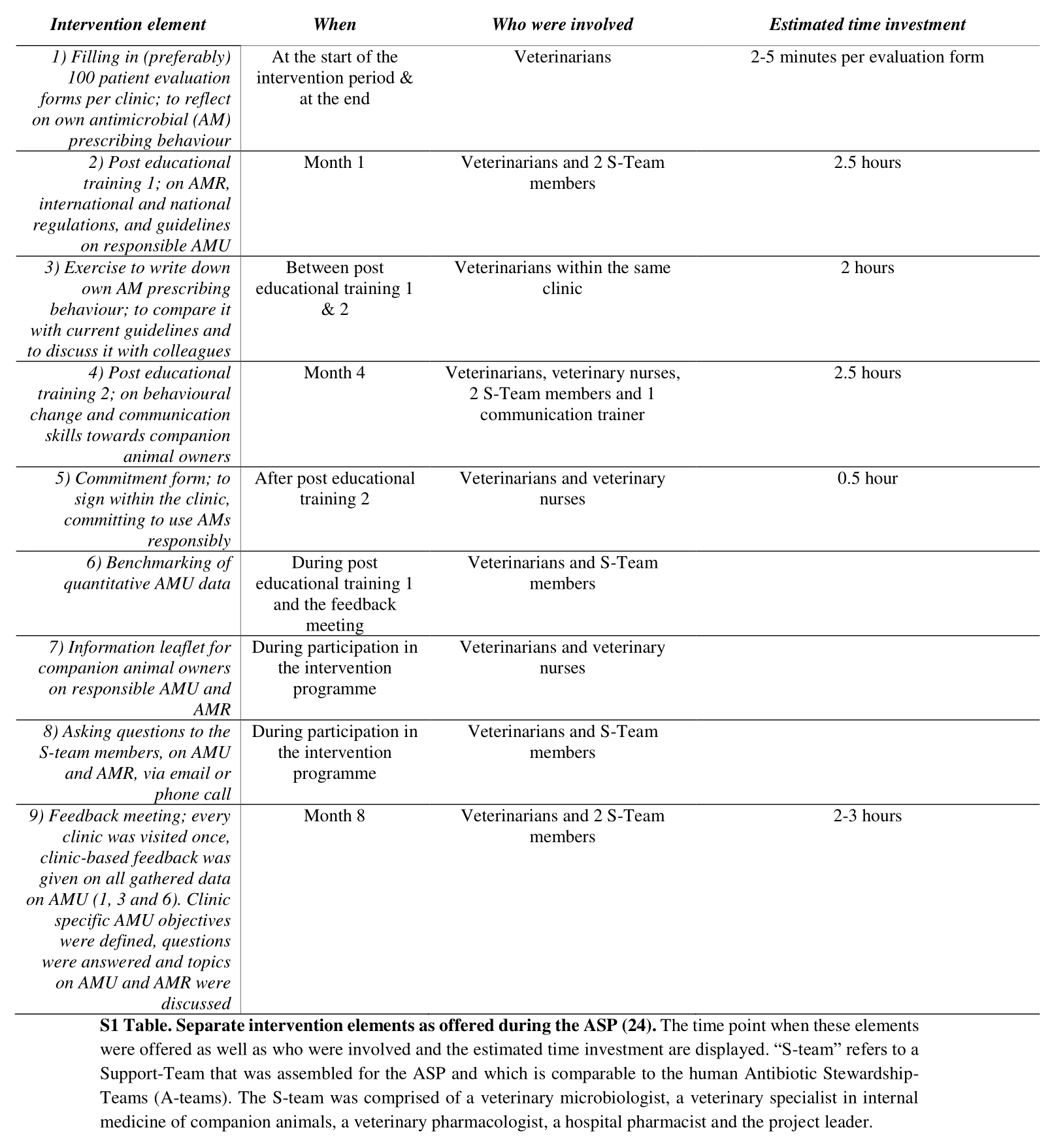

Supplement: S1 Table — The time point when these elements were offered as well as who were involved and the estimated time investment are displayed. “S-team”refers to a Support-Team that was assembled for the ASP and is comparable to the human Antibiotic Stewardship-Teams (A-teams). The S-team was comprised of a veterinary microbiologist, a veterinary specialist in internal medicine of companion animals, a veterinary pharmacologist, a hospital pharmacist and the project leader. (TIF) [file pone.0283956.s001.tif]

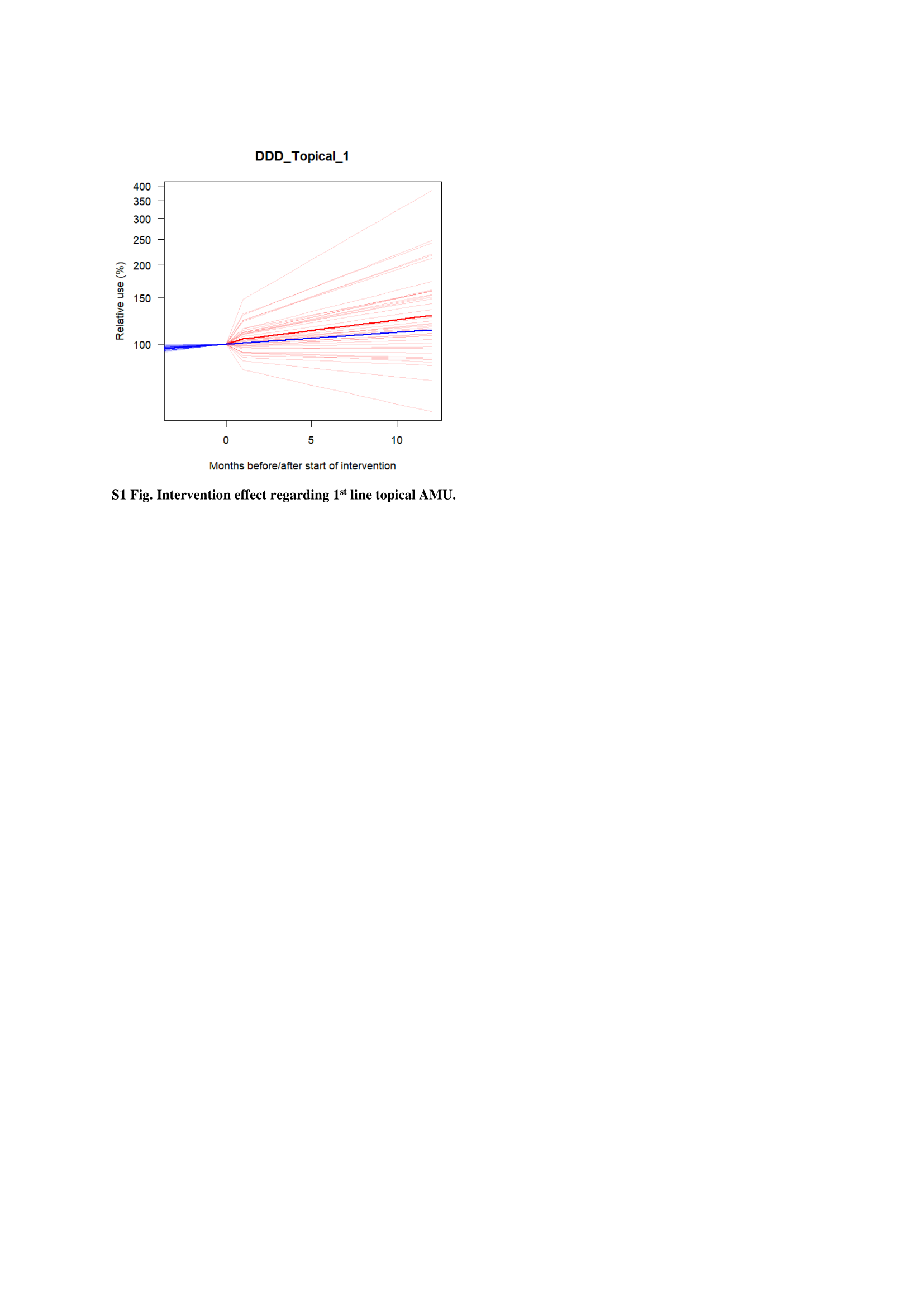

Supplement: S1 Fig — (TIF) [file pone.0283956.s002.tif]

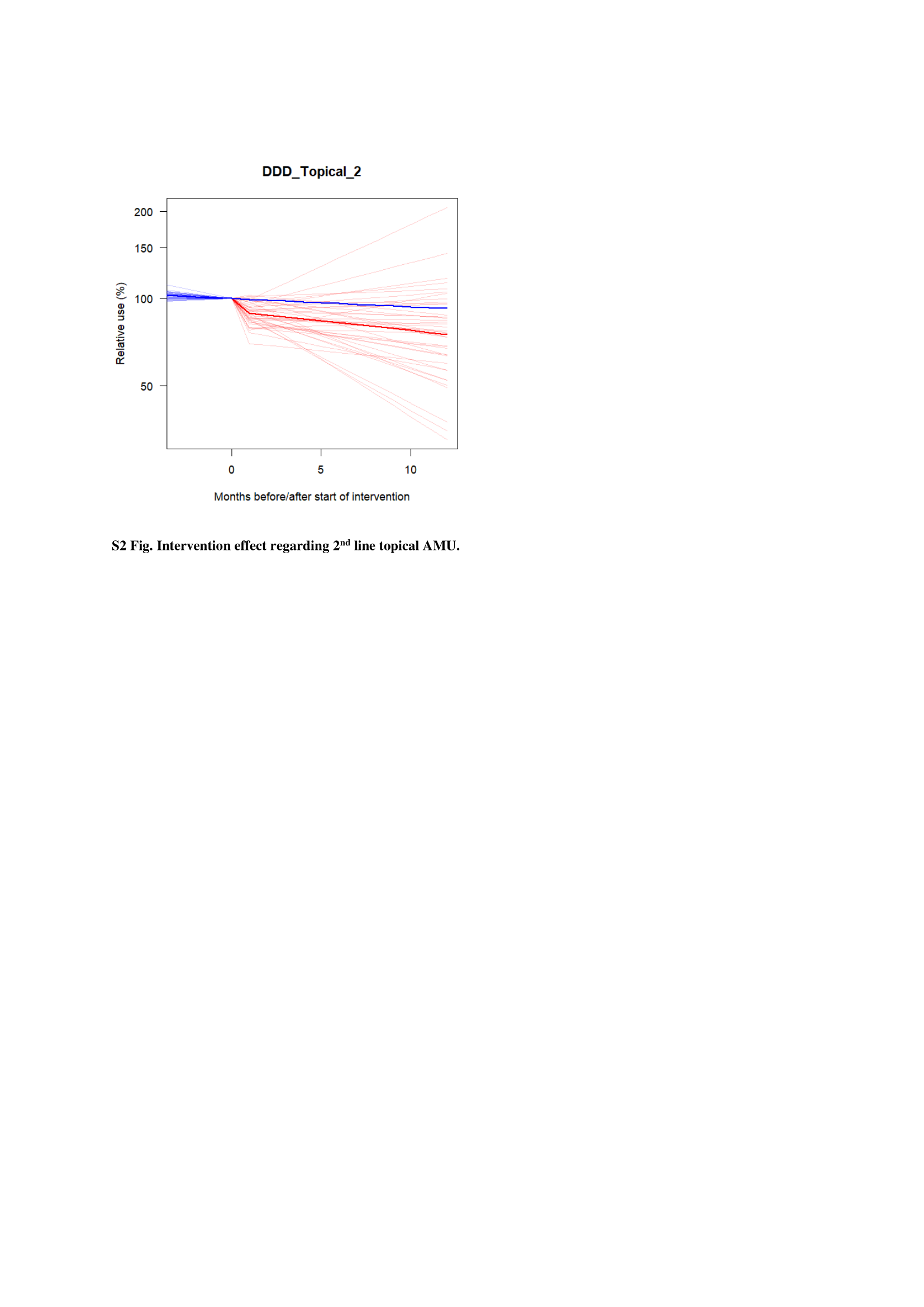

Supplement: S2 Fig — (TIF) [file pone.0283956.s003.tif]

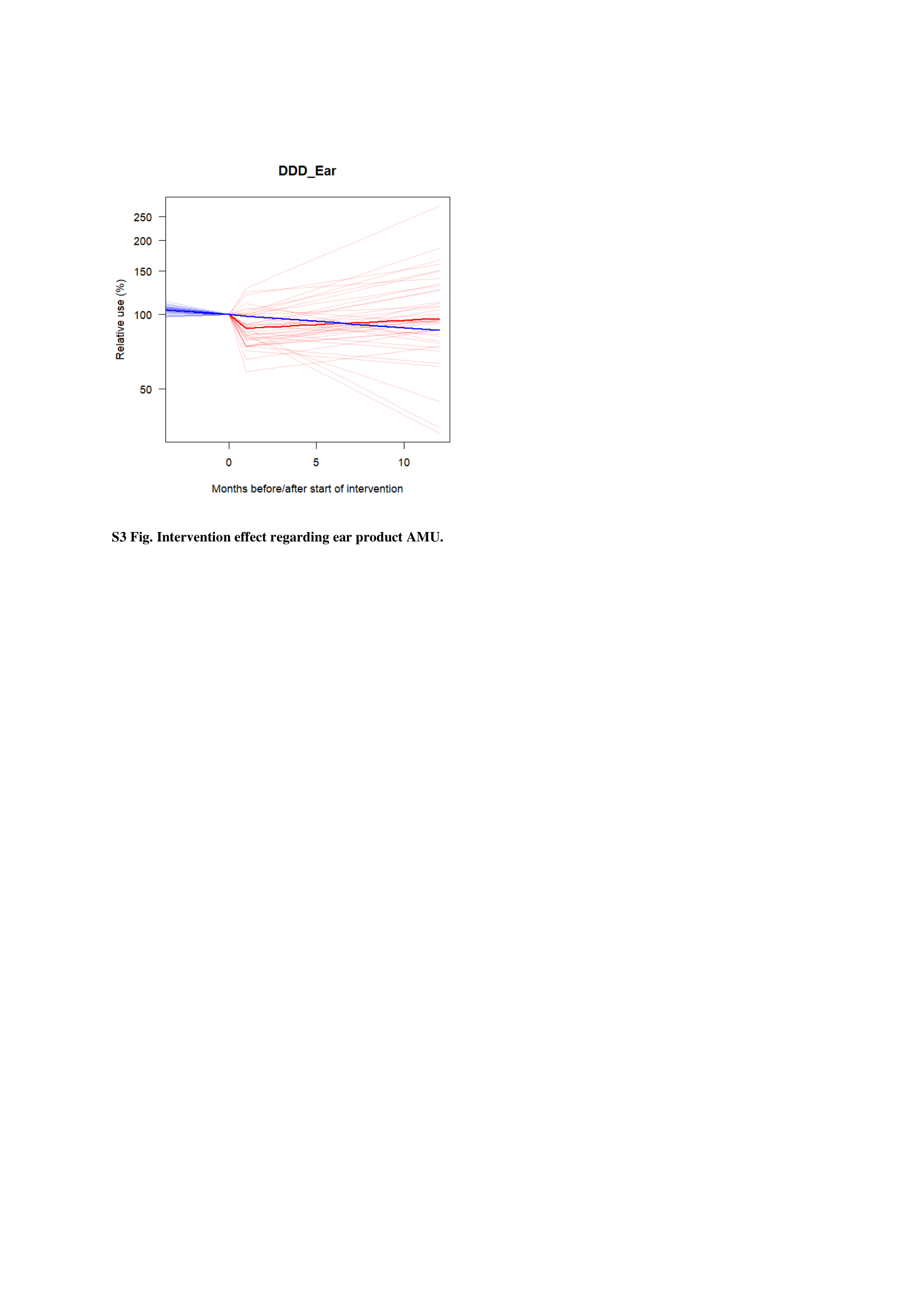

Supplement: S3 Fig — (TIF) [file pone.0283956.s004.tif]

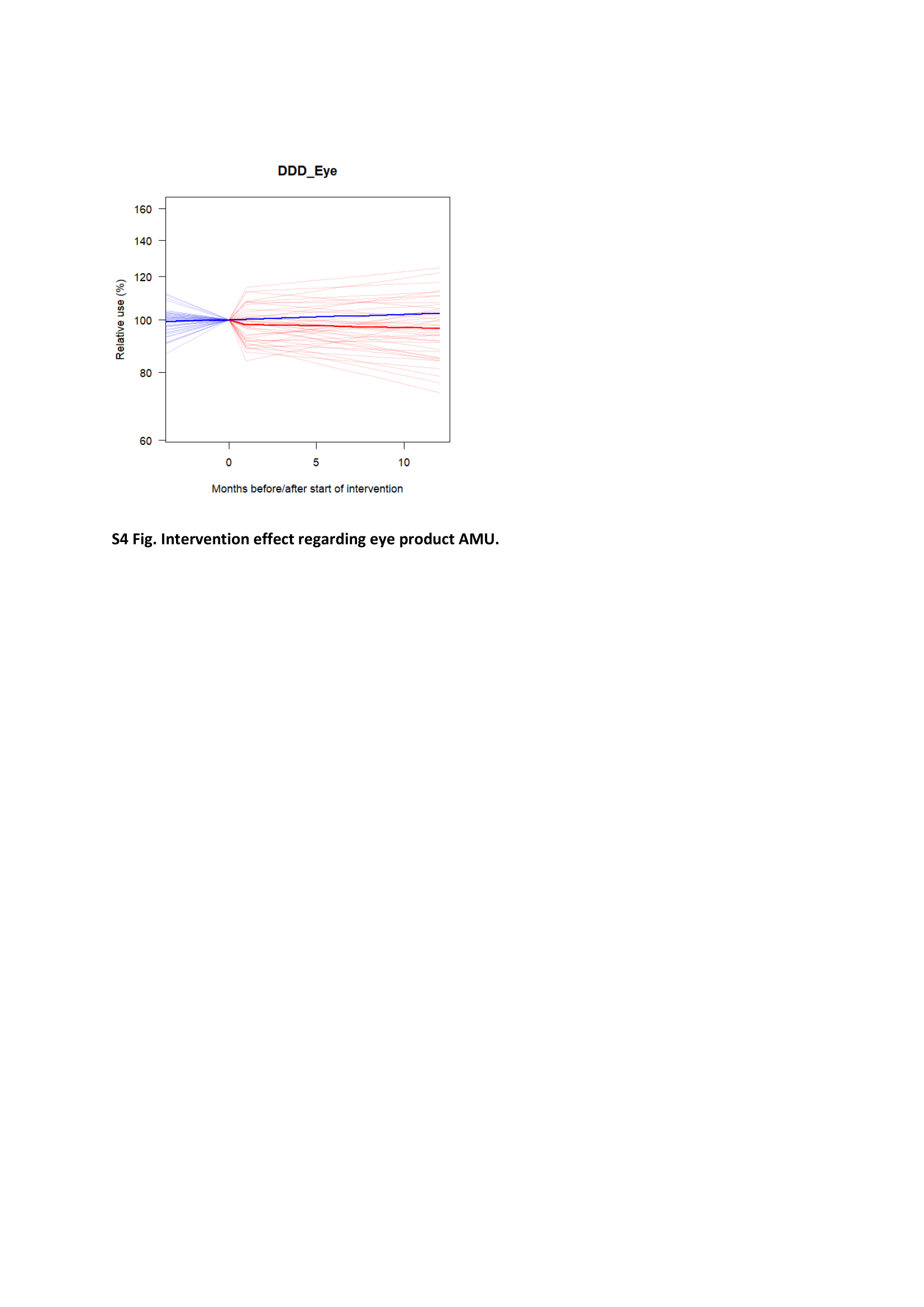

Supplement: S4 Fig — (TIF) [file pone.0283956.s005.tif]

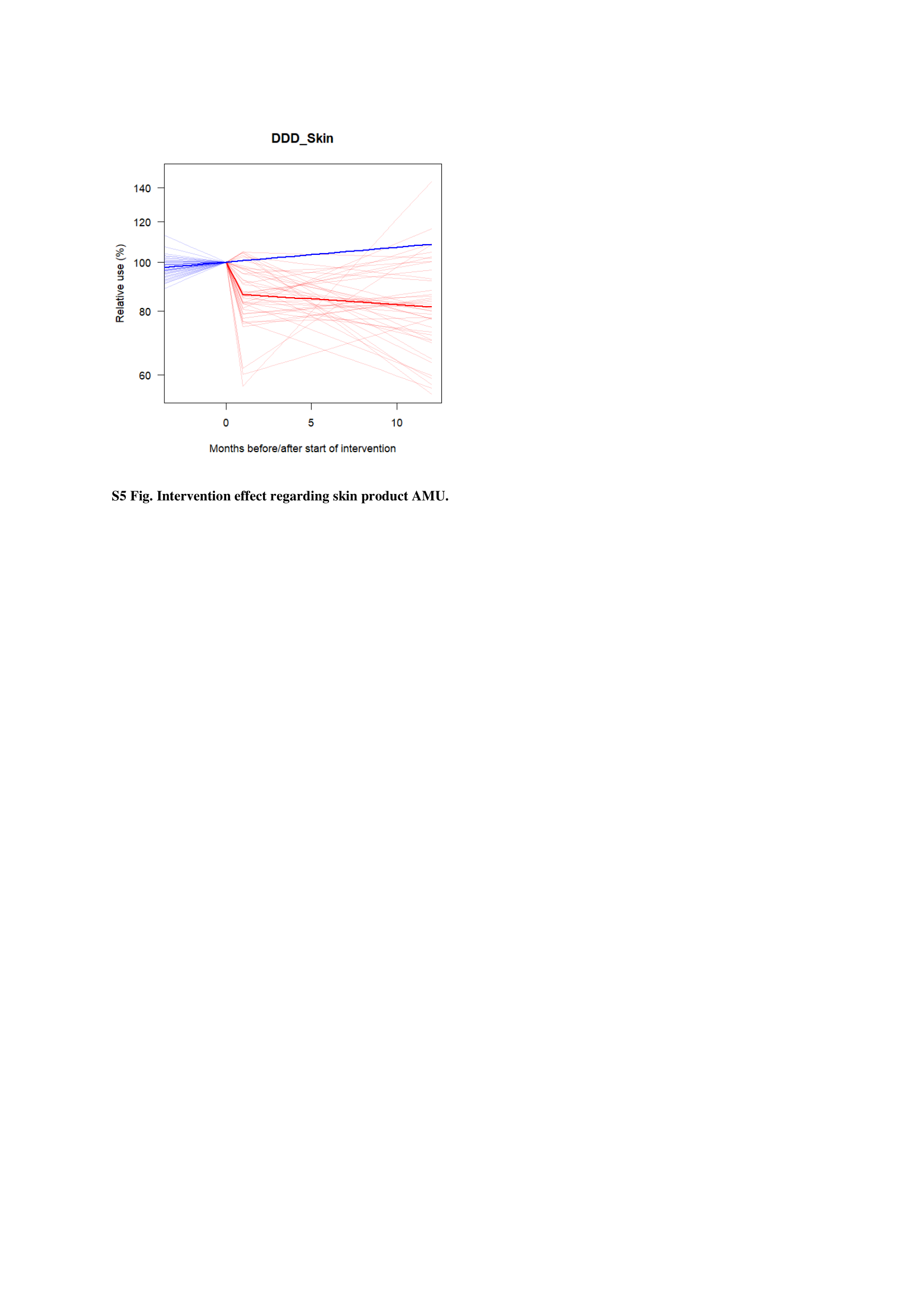

Supplement: S5 Fig — (TIF) [file pone.0283956.s006.tif]
